# COVID-19-Related Bilateral Avascular Necrosis of the Femoral Head

**DOI:** 10.7759/cureus.44034

**Published:** 2023-08-24

**Authors:** Adesuwa Inneh, Kayla Martinez, Juleen Elizee, Malika Ganguli, Aydin Turan

**Affiliations:** 1 Urology, Ross University School of Medicine, Pontiac, USA; 2 Surgery, Ross University School of Medicine, Pontiac, USA; 3 Pediatrics/Internal Medicine, Ross University School of Medicine, Pontiac, USA; 4 Internal Medicine, Ross University School of Medicine, Pontiac, USA; 5 Internal Medicine, Trinity Health Oakland Hospital, Pontiac, USA

**Keywords:** bone necrosis, avascular necrosis of the hip, steroid use, orth. surg. consultant, avascular osteonecrosis, covid 19

## Abstract

Osteonecrosis is a pathologic process that involves focal bone infarction and death of bone tissue caused by trauma, infections, autoimmune conditions, and chronic steroid use; however, most cases go undiagnosed. The link between bilateral osteonecrosis and coronavirus disease 2019 (COVID-19) infections has not been fully investigated. This is the case of a 42-year-old Caucasian woman who presented to the emergency department for bilateral hip pain, which started three months prior. Initially, the pain was mild; however, her symptoms worsened, causing her to have difficulty ambulating. Co-incidentally she tested positive for COVID-19 10 days after the onset of pain. She denied any lower-extremity numbness, weakness, and loss of bowel or bladder function. X-ray of the hips showed significant sclerosis of bilateral femoral heads and acetabula, indicating avascular necrosis. She was given ketorolac injection intramuscularly for analgesia and remained in stable condition. Upon discharge, she was given a referral to orthopedic surgery for bilateral total hip arthroplasty. Atraumatic osteonecrosis of the femoral head can be caused by multiple etiologies, including exposure to medications, post-transplantation procedures, trauma, and hypercoagulable states. This condition is likely due to poor angiogenesis after an infarct, causing a domino effect of bone demineralization, trabecular thinning, and cortical collapse. A literature search demonstrated prior cases of unilateral femoral head necrosis associated with COVID-19 infection and steroid use. There have been no cases of bilateral osteonecrosis of the femoral head reported without long-term steroid use. Considering the disease severity in both hips and limited steroid use (only five days of prednisone), other common etiologies were sought and were ruled out. In our patient, the only event that was related to her initial onset of hip pain was a COVID-19 infection. We suggest a relationship between COVID-19 infection and avascular necrosis given the rapid progression of the disease. We acknowledge that this presentation of bilateral osteonecrosis is rare and warrants further investigation. More research should be performed to establish a tenable relationship between COVID-19 infection and osteonecrosis, with and without the use of steroids.

## Introduction

Avascular necrosis (AVN), also referred to as osteonecrosis, is a pathologic process that involves focal bone infarction and death of bone tissue caused by trauma, infections, autoimmune conditions, and chronic steroid use. Commonly affecting the epiphysis of long bones and pelvic bones, UpToDate reports that the incidence of AVN is 10-20,000 in the United States [1]. However, most cases go undiagnosed. The link between bilateral AVN and COVID-19 infection has not been fully investigated.

## Case presentation

This is the case of a 42-year-old Caucasian woman who presented to the emergency department for bilateral hip pain. The patient reported that the pain in her hips started approximately 2-3 months ago. Initially, her pain was mild but progressively worsened over two months. Coincidentally she tested positive for SARS COVID-19 at that time. Due to severe pain, she was unable to ambulate. She denied any lower-extremity numbness, weakness, or loss of bowel or bladder function. A summary of the patient's abnormal lab values can be seen in Table 1.

**Table 1:** Patient's Abnormal Lab Findings The patient’s WBC, HIV, UA, and pregnancy tests were all unremarkable and within normal limits. WBC, white blood cell; HIV, human immunodeficiency virus; UA, urinalysis.

Labs and Normal Reference Range	Normal Values	Patient’s Values
C-Reactive Protein	0.3-1.0 mg/dL	0.6 mg/dL
Vitamin D	>20 ng/mL	27 ng/mL
Erythrocyte Sedimentation Rate	0-20 mm/h	16 mm/h
D-Dimer	0-250 mg/L FEU	237 mg/L FEU
Antinuclear Antibody Titer	Negative if <1:80	Positive with >1:80

**Figure 1:**
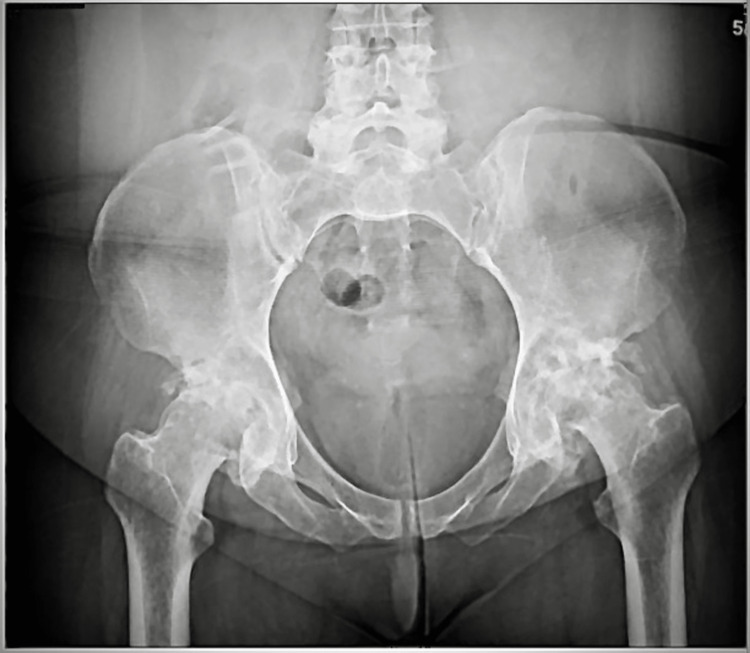
Bilateral Hip X-ray Plain radiograph suggesting osteonecrosis of the femoral head with evident sclerosis and joint space narrowing bilaterally.

**Figure 2:**
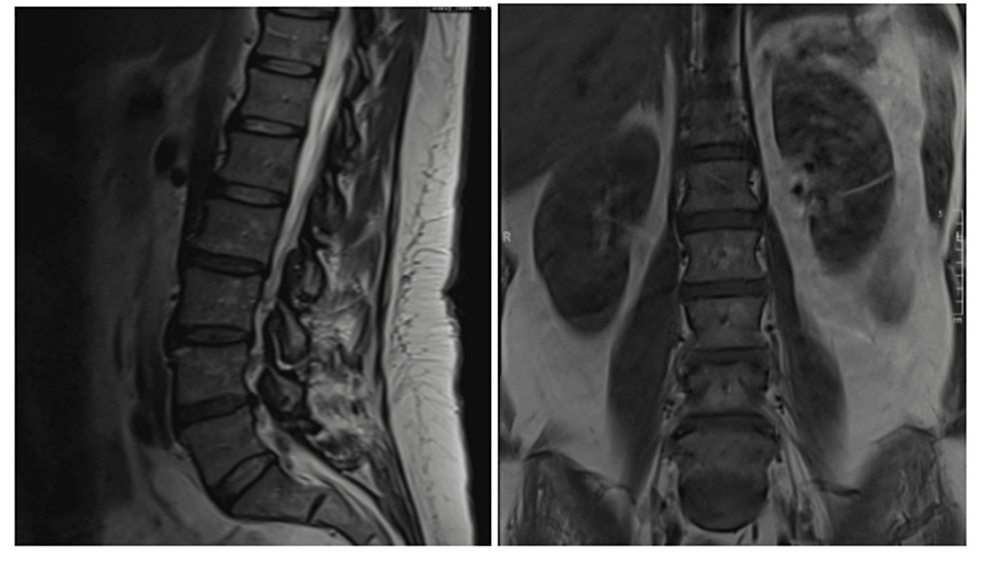
MRI of Lumbar Spine Without Contrast Imaging demonstrating mild lumbar spondylosis, moderate disc degeneration at L4-L5, mild disc degeneration at L2-L3 and L3-L4, no traumatic injury, fracture, subluxation, or suggestion of acute process of infection. MRI, magnetic resonance imaging.

X-ray of bilateral hips (Figure 1) showed significant sclerosis of bilateral femoral heads and bilateral acetabula, indicating AVN. Magnetic resonance imaging of the lumbar spine (Figure 2) showed mild lumbar spondylosis and moderate radiculopathy with moderate spinal stenosis at the level of L4-L5. Mass effect was also noted to the right L5 nerve root due to spinal canal stenosis. 

Our patient has a history of chronic back pain, for which she sees pain management. In addition to being given Toradol IM for faster analgesic relief in the office, her physician prescribed a Medrol dose pack PO taper for five days. Initially, her pain subsided from these interventions. However, looking retrospectively now, we believe these medications could have possibly delayed our patient from seeking more extensive medical care to diagnose bilateral AVN. 

## Discussion

Atraumatic AVN of the femoral head can be caused by multiple etiologies, including exposure to medications, post-transplant, trauma, hypercoagulability, and heritable disorders. Lang et al. found that the pathophysiology and natural history of this condition are not fully understood, partly due to the asymptomatic nature of early disease [2]. It is likely that the disease progresses due to poor angiogenesis after an infarct, causing a domino effect of demineralization, trabecular thinning, and cortical bone collapse.

The onset of AVN in COVID-19 is poorly documented; however, select articles suggest its prevalence. Dhanasekararaja et al. reported 22 cases of AVN in patients recovering from COVID-19. Patients showed symptoms in a time frame ranging from three weeks to three months, similar to a case series by Agarwala et al. with three patients showing symptoms 45-57 days after confirmation of COVID-19 infection [3,4]. Perhaps, this suggests a guideline for clinicians to have a higher suspicion of AVN in post-COVID patients with complaints of hip pain. A literature search demonstrated case reports of unilateral femoral head necrosis associated with COVID-19 infection and steroid use. However, there have been no cases of bilateral AVN of the femoral head per this author’s literature review.

Theorized pathophysiology is due to the role of systemic steroids used to manage the inflammatory cytokine response during infection [3]. Steroid use is a known risk factor for the development of AVN. The risk may be amplified due to the known induction of a hypercoagulable state during COVID-19. Beck et al. reported that interactions between pro-inflammatory cytokines may cause increased platelet aggregation and endothelial cell damage [5]. Tang et al. found that similar emergences of AVN were seen during the early 2000's SARS epidemic, prompting articles cautioning clinicians to limit corticosteroid use only in critically ill or septic patients undergoing treatment for COVID-19 [6]. Zhao et al. suggest a dose-response gradient to steroid and ONFH (osteonecrosis of the femoral head) development, where doses less than 5 mg provide the lowest risk and doses more than 10 mg have a high risk [7]. This suggests that our patient’s use of a Medrol dose pack with a max dose of 24 mg every 24 hours has a relatively low risk of ONFH development secondary to exogenous steroid use. 

COVID-19 has been theorized to cause worsening or abnormal inflammation in hospitalized patients. Being that our patient was COVID-19 positive at the time of her illness, we acknowledge that her presentation of AVN could have been impacted or superimposed by this viral illness. Dugue et al. described COVID-19-associated coagulopathy (CAC) as an emerging hallmark associated with macrovascular and microvascular complications in COVID-19-positive patients [8]. CAC-induced tissue necrosis is not a well-understood mechanism but is believed to be linked to hypercoagulability, overwhelming viral pathologic effects, and immune system hyperactivity. CAC is an acute acquired state defined as hypercoagulability, with elevated D-dimer and fibrinogen, prolonged partial thromboplastin time (PTT), associated with acute limb ischemia, thrombosis, and digital gangrene. Dugue et al. reported a case of extensive tissue necrosis in a COVID-19-positive patient, after a successful above-elbow right arm amputation. Perhaps the hypercoagulable state seen in numerous COVID-19-positive patients predisposes them to thrombotic disease, ultimately increasing the risk of necrosis to tissues [8].

The two main differential diagnoses considered in this case after a thorough history review, physical examination, and investigations were steroid use and idiopathic cause. However, considering the disease severity in bilateral hips, with limited steroid use by the patient, as well as negative history findings supporting medication complications, other etiologies were sought. Although during her hospital stay she was found to have a positive antinuclear antibody (ANA), her titer was at the cusp of normal (1:80). According to Taeschler et al., an elevated ANA could be due to numerous etiologies, and not just autoimmune diseases, such as a viral illness like her previous COVID-19 diagnosis [9]. The only event related to the initial onset of hip pain was the COVID-19 infection.

## Conclusions

Based on a thorough clinical investigation, there is not a clear etiology for this 42-year-old female presenting with bilateral hip pain with a positive COVID-19 test demonstrating bilateral AVN. We suggest a relationship between COVID-19 infection and AVN given the rapid progression of disease and recent COVID-19 infection. We highlighted pertinent negatives pointing away from steroid-induced AVN; our patient had limited use of corticosteroids during this time, and it is unlikely that this medication played a role in the patient’s pathophysiology of bilateral AVN. We acknowledge that additional research needs to be performed to establish a concrete relationship between COVID-19 infection and AVN of the femoral head. Concurrently, we acknowledge that additional research should be conducted on short-term steroid use during a COVID-19 infection in relation to AVN. We assessed the known risk factors of AVN such as extensive alcohol use and hypercoagulable states seen in malignancy or autoimmune diseases and analyzed our patient’s medical history. Our patient admitted to only having four alcoholic beverages per year, making her alcohol use an unlikely risk factor for this disease. Our patient was never diagnosed with cancer or autoimmune diseases, making hypercoagulability from these diseases an unlikely risk factor for her AVN. After interviewing our patient and performing an extensive medical record review, no additional autoimmune testing was conducted as our patient was lost to follow-up. We acknowledge that the patient could have been in a hypercoagulable state due to inflammation from COVID-19. Imaging studies, specifically X-ray of the pelvis, demonstrated necrosis of bilateral femoral heads. MRI of the lumbar spine showed chronic degeneration changes, as stated in the patient’s past medical history. Autoimmune, traumatic, and steroid etiologies were ruled out. This report suggests a link between COVID-19 infection and osteonecrosis, specifically bilateral osteonecrosis as seen in this patient.
